# Supplementary material for: Targeting the conserved active site of splicing machines with specific and selective small molecule modulators
Source: Nat Commun. 2024 Jun 19;15:4980. doi: 10.1038/s41467-024-48697-0 (PMC11187226; doi:10.1038/s41467-024-48697-0)
Supplement: Supplementary file 1 — Supplementary Information File [file 41467_2024_48697_MOESM1_ESM.pdf]

SUPPLEMENTARY INFORMATION FILE FOR:

Targeting the conserved active site of splicing machines with specific and selective small molecule modulators

Ilaria Silvestri[1,2,†], Jacopo Manigrasso[1, ‡], Alessandro Andreani[1], Nicoletta Brindani[1], Caroline Mas[3], Jean-Baptiste Reiser[4], Pietro Vidossich[1], Gianfranco Martino[1], Andrew A. McCarthy[2], Marco De Vivo[1,*], Marco Marcia[2,*]

[*]To whom correspondence should be addressed. E-mail: marco.devivo@iit.it; mmarcia@embl.fr

This Supplemental File includes:

- Supplemental Figures S1-S29
- Supplemental Tables S1-S2
- Supplemental References

SUPPLEMENTAL MATERIAL

Supplemental Figures

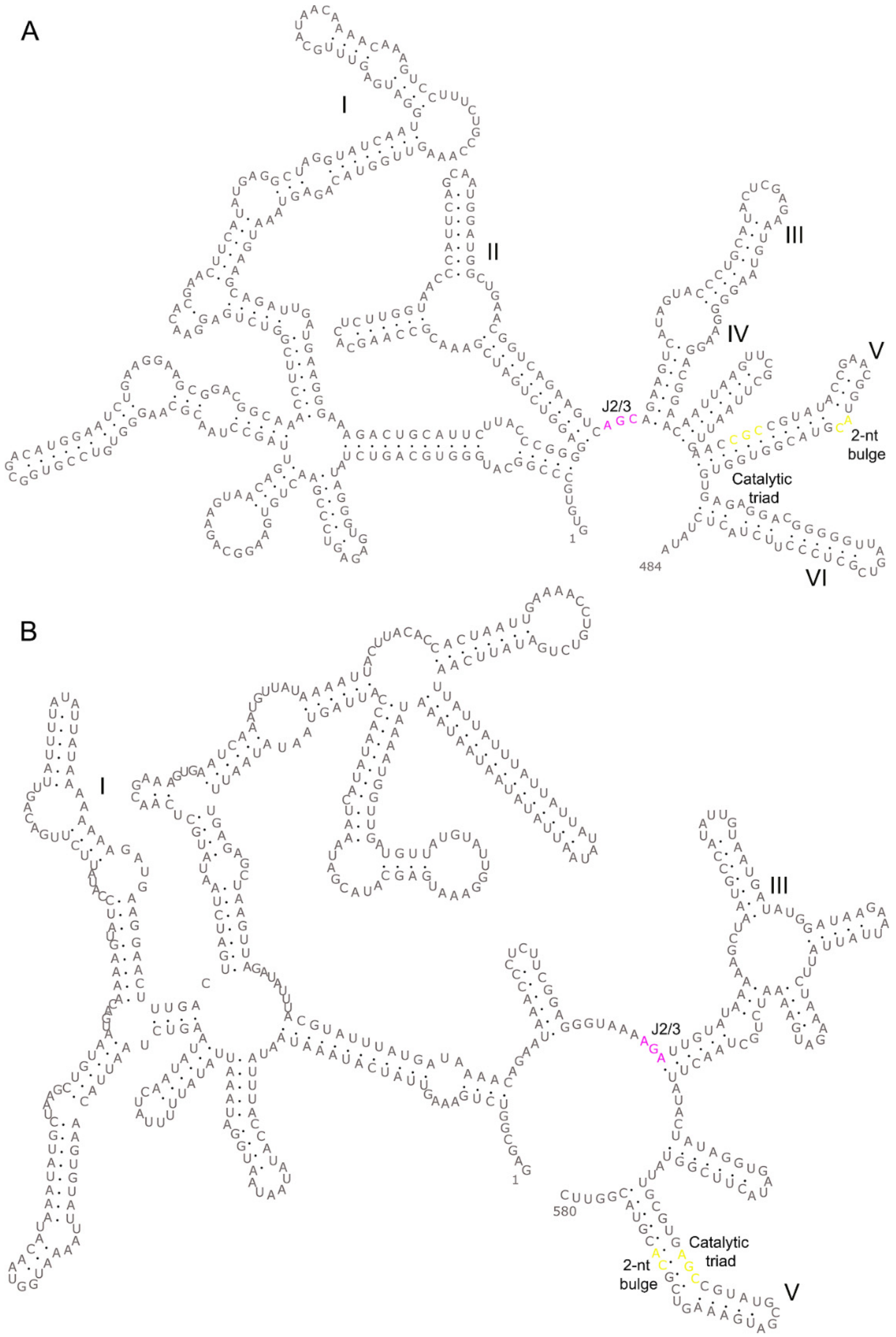

Figure S1. Secondary structure map of group II introns used in this work. (A) Secondary structure map of the I1 group IIC intron of *O. iheyensis* used in this work. The different domains are indicated with Roman numerals. The main elements of the catalytic site are indicated with different colors, i.e. the J2/3 junction in magenta, the catalytic triad and the 2-nucleotide bulge in yellow. **(B)** Secondary structure map of the D135 ai5γ group IIB intron of *S. cerevisiae* used in this work. The different domains are indicated with Roman numerals. The main elements of the catalytic site are indicated with different colors, i.e. the J2/3 junction in magenta, the catalytic triad and the 2-nucleotide bulge in yellow.

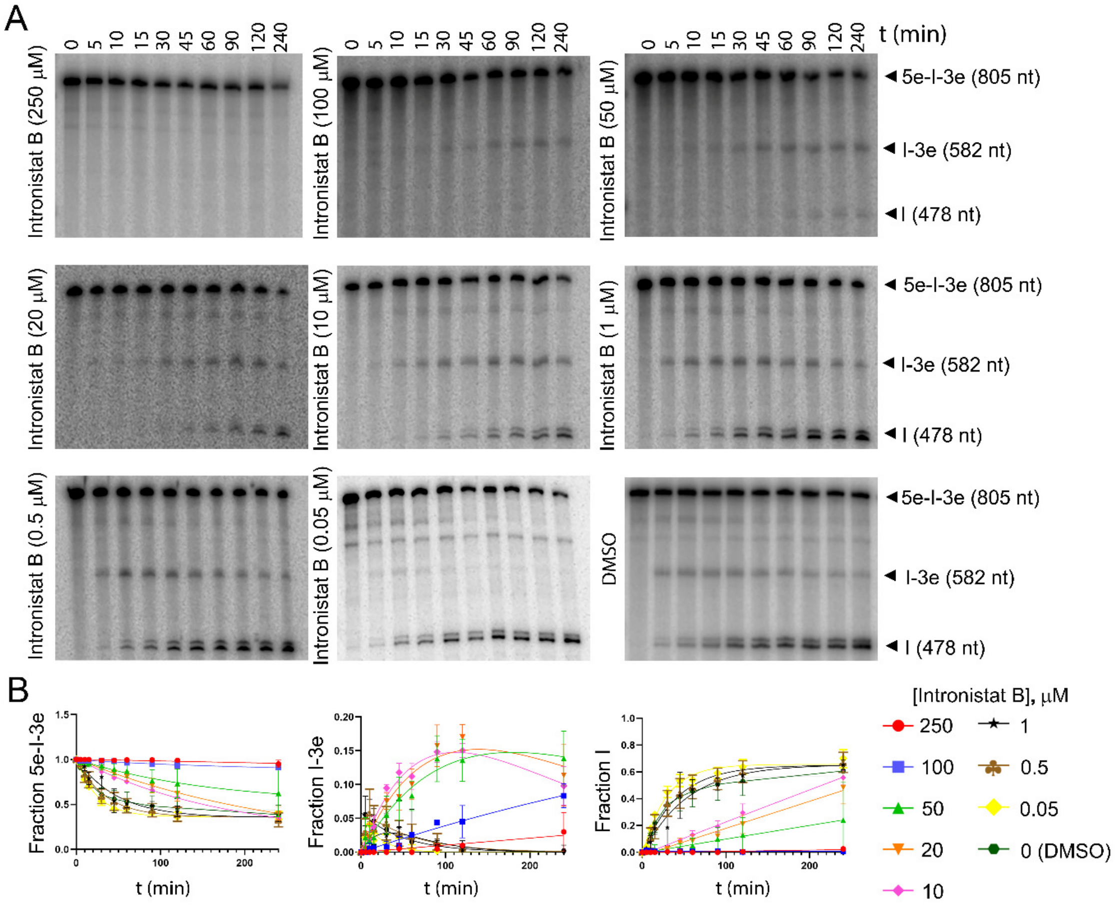

Figure S2. Kinetics of splicing inhibition. (A) Splicing kinetics in the presence of Intronistat B at all concentrations tested in this study. The relative rate constants are listed in **Table S1**. Precursors are indicated as 5e-I-3e (nt length in parenthesis). Intermediate (I-3e) and linear intron (I) migrate as double bands because of cryptic cleavage sites, as explained previously[1,2]. **(B)** Evolution of the populations of precursor (5e-I-3e, left panel), intermediate (I-3e, middle panel), and linear intron (I, right panel) over time. Error bars represent standard errors of the mean (s.e.m.) calculated from at least n = 3 independent experiments. Source data are provided as a Source Data file.

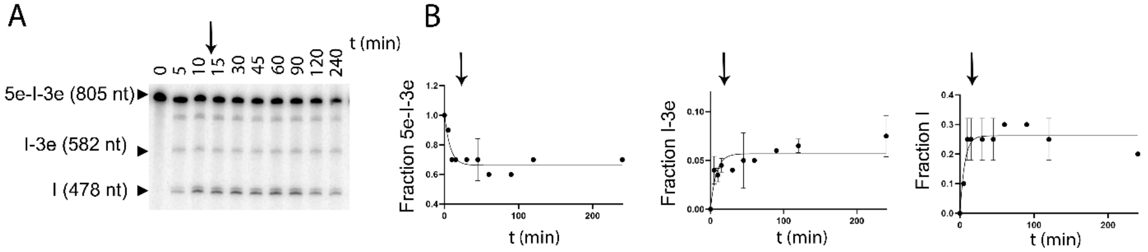

Figure S3. Intronistat B targets folded, active group II introns. (A) Representative splicing kinetics in the presence of 250 µM intronistat B, which is added 10 min after the start of the splicing reaction (black arrow). **(B)** Evolution of the populations of precursor (5e-I-3e, left panel), intermediate (I-3e, middle panel), and linear intron (I, right panel) over time. The black arrows indicate the time when intronistat B was added to the reaction. Source data are provided as a Source Data file.

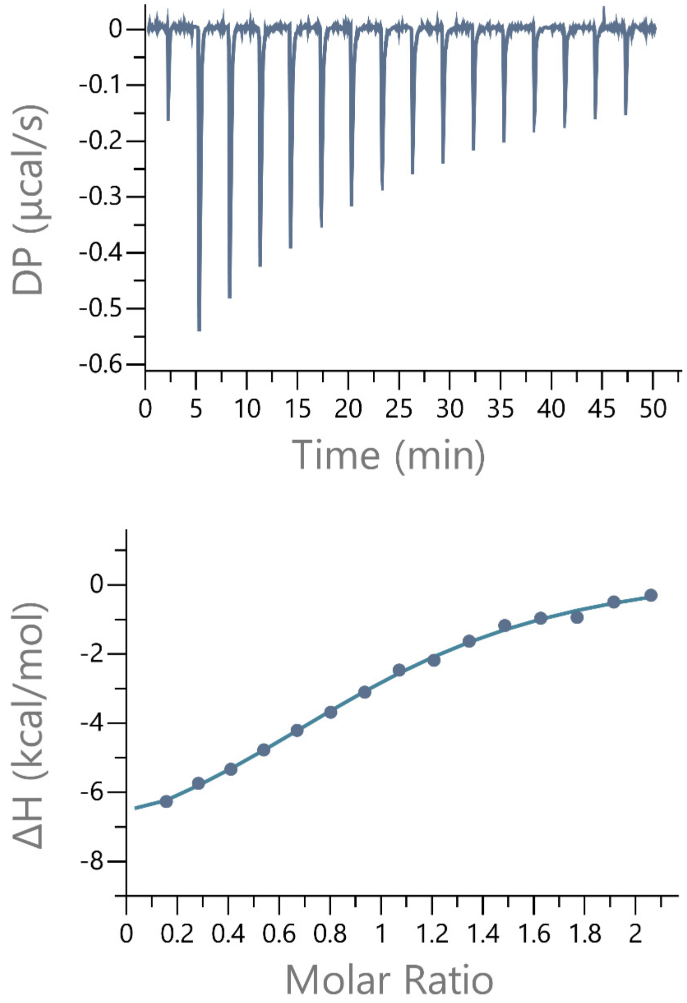

Figure S4. Isothermal titration calorimetry (ITC). (A) Raw data of the heat pulses resulting from titration of Oi1-5 group II intron (30 µM) in the calorimetric cell with intronistat B (600 µM). **(B)** Integrated heat pulses, normalized per mol of injectant as a function of the molar ratio (ligand/intron concentration).

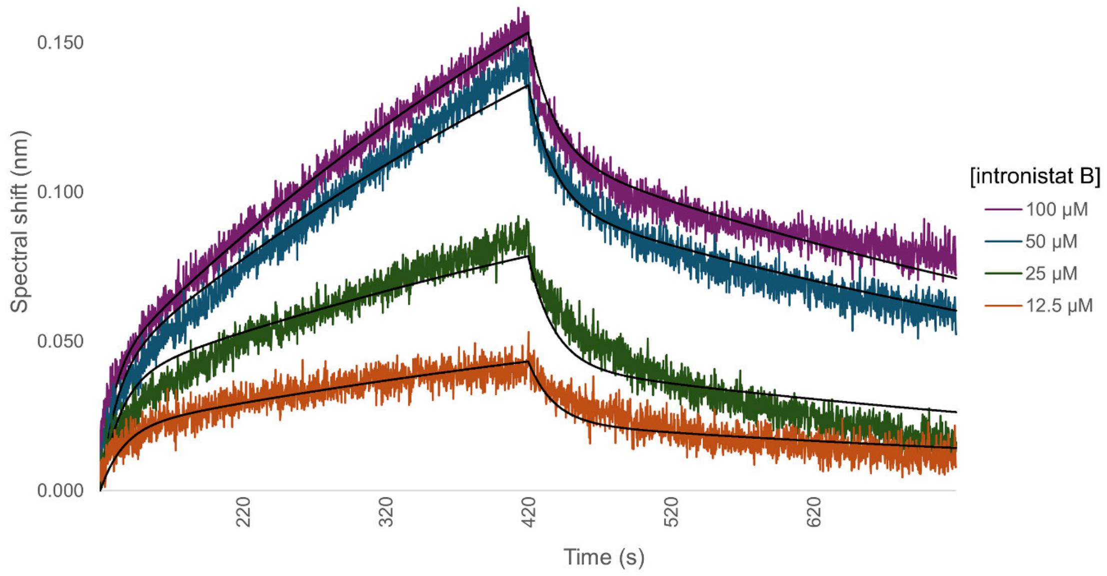

Figure S5. Biolayer interferometry (BLI). BLI sensorgrams depicting the direct and real-time binding of intronistat B to Oi1-5 group II intron. The binding curves were used to determine kinetics rate constants and by globally fitting the rate equation for 2:1 heterogeneous kinetics. Fittings are reported as black lines.

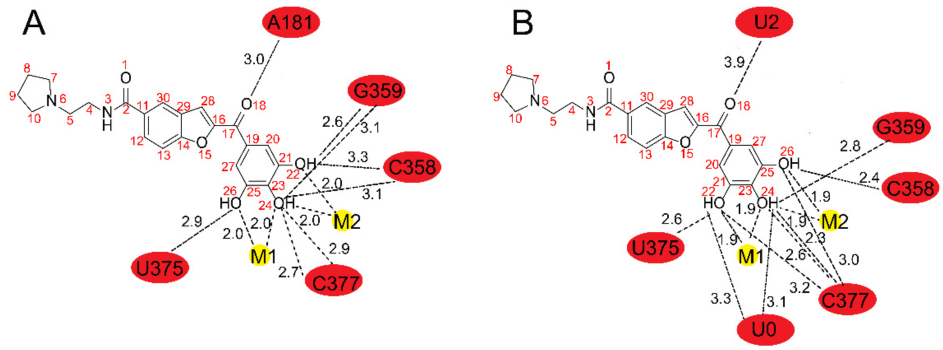

Figure S6. Intronistat B chemical structure and interactions with the active site of group II introns. (A) Intronistat B and its interactions with the group II intron in the exon free state (related to **Figure 2**). **(B) I**ntronistat B and its interactions with the group II intron in the exon bound state (related to **Figure 4**). Black dotted lines indicate the interactions between the atoms of intronistat B and the group II intron active site nucleotides (red circles) or catalytic metals (yellow circles). The weak contact between intronistat B O18 and the U2 O4' atom is indicated by a gray dotted line. Distances are indicated in Å next to each dotted line.

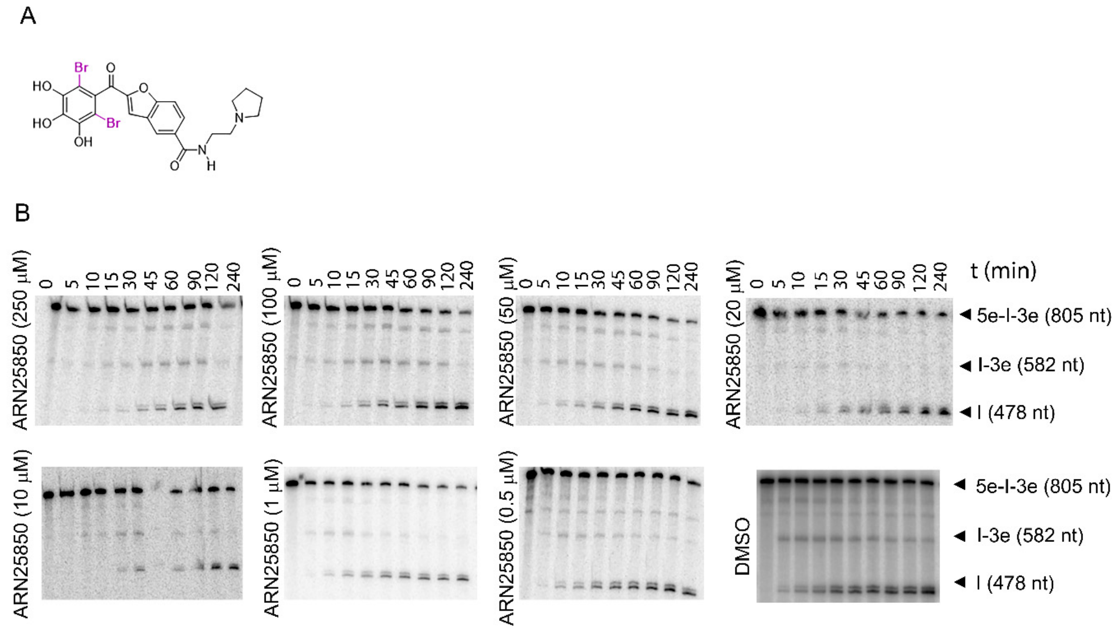

Figure S7. Group II intron inhibition by the di-brominated intronistat B derivative, ARN25850. (A) Chemical structure of ARN25850. **(B)** Representative splicing kinetics in the presence of different concentrations of ARN25850. The relative rate constants are listed in **Table S1**. Source data are provided as a Source Data file.

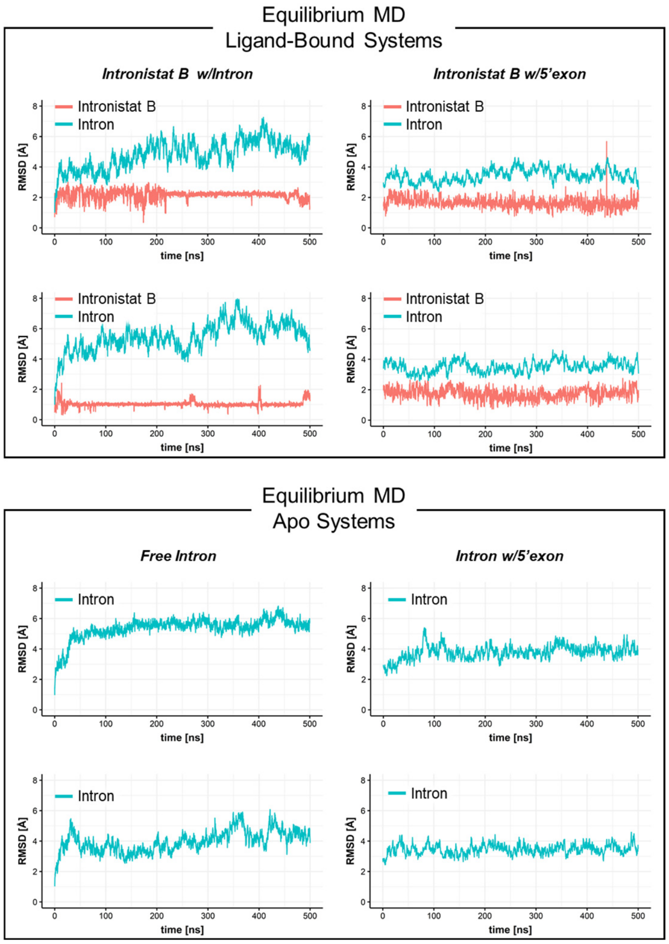

Figure S8. MD simulations of the free and 5'exon-bound intron. On the top four panels, the RMSD value of the intron (cyan) and the intronistat B (red) is reported as a function of the simulation time for the free (left) and 5'exon-bound (right) system. On the bottom four panels, the RMSD of the free (left) and 5'exon-bound (right) intron is reported.

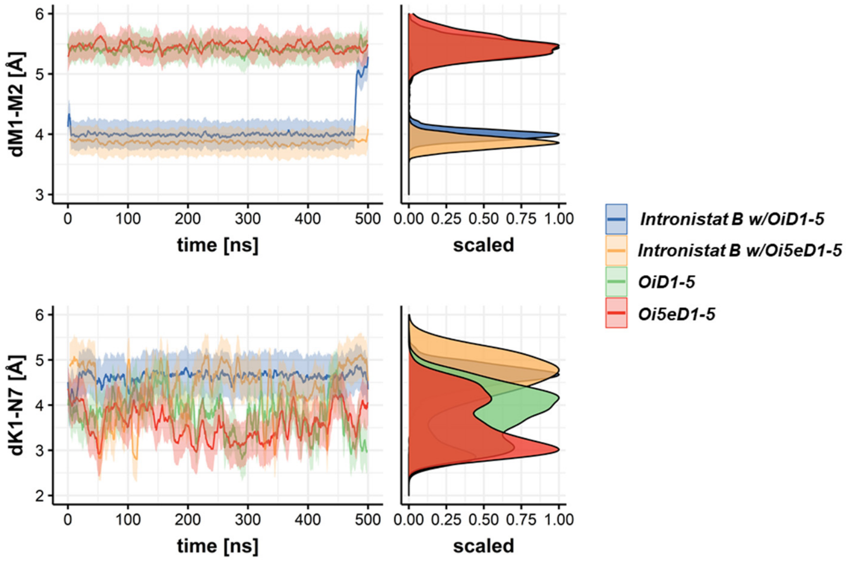

Figure S9. Binding of splicing modulators alters the functional dynamics of intron's catalytic features. The Figure reports MD replicas in support of simulations shown in **Figure 3**. The standard deviation is shown as a shaded area.

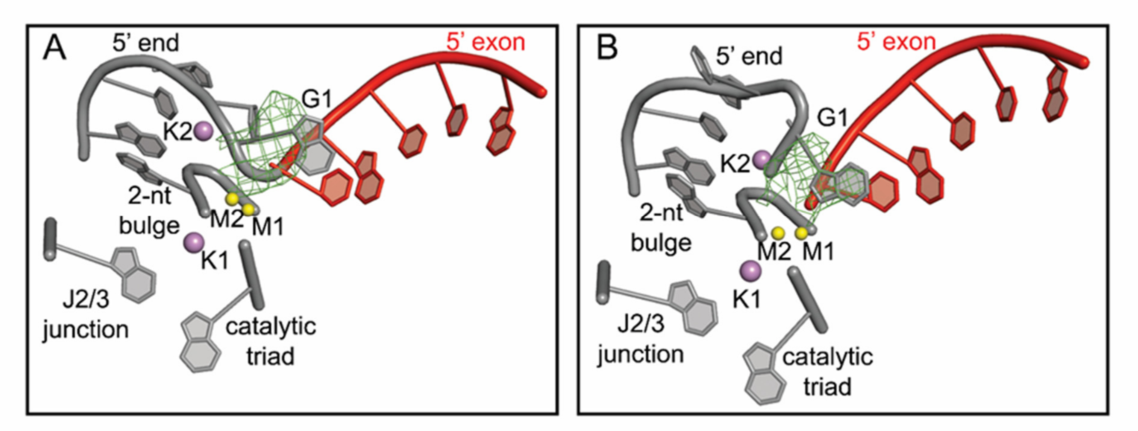

Figure S10. The 5’-splice junction outcompetes intronistat B in the active site. (A) Crystal structure of Oi5eD1-5 in the pre-catalytic stage in the presence of Ca^{2+} (yellow spheres), K^{+} (purple spheres) and intronistat B. **(B)** Crystal structure of Oi5eD1-5 in the post-catalytic stage in the presence of Mg^{2+} (yellow spheres), K^{+} (purple spheres) and intronistat B. The F_o-F_c electron density omit map calculated by omitting intron residue G1 and contoured at 3σ is represented as a green mesh in both panels. The 5’-exon is represented as red sticks in both panels. Intronistat B is not bound to the active site under these conditions.

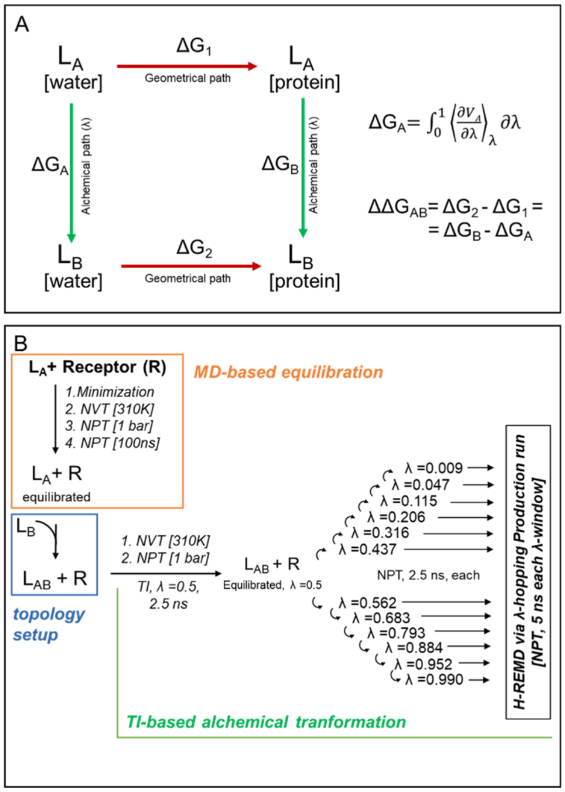

Figure S11. Thermodynamic integration-based alchemical free energy calculations. (A) The thermodynamic cycle at the basis of the estimations of relative binding free energy. Additionally, the equation for deriving the ΔG along every alchemical path, as well as that for deriving the ΔΔG is reported. **(B)** The protocol for the alchemical free energy calculations.

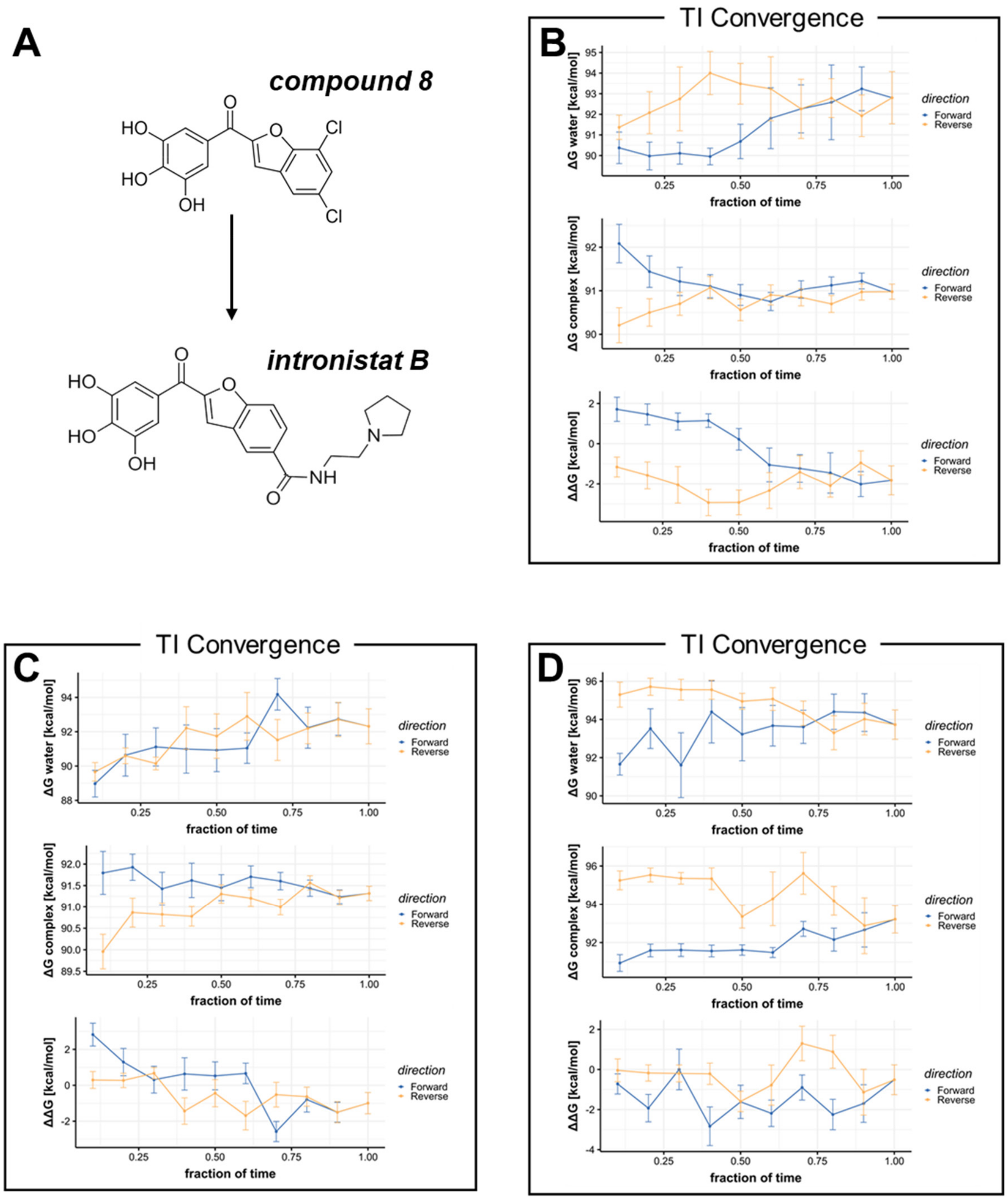

Figure S12. Alchemical free energy calculations for compound 8 and intronistat B bound to the intron in complex with the 5'-exon. **(A)** The 2D structures of the compounds are reported. The convergence of the forward (blue) and reverse (yellow) estimates of the ΔG of the ligands in water and as bound to the receptor, as well and their ΔΔG is reported of each of three simulations replica **(B-D)**.

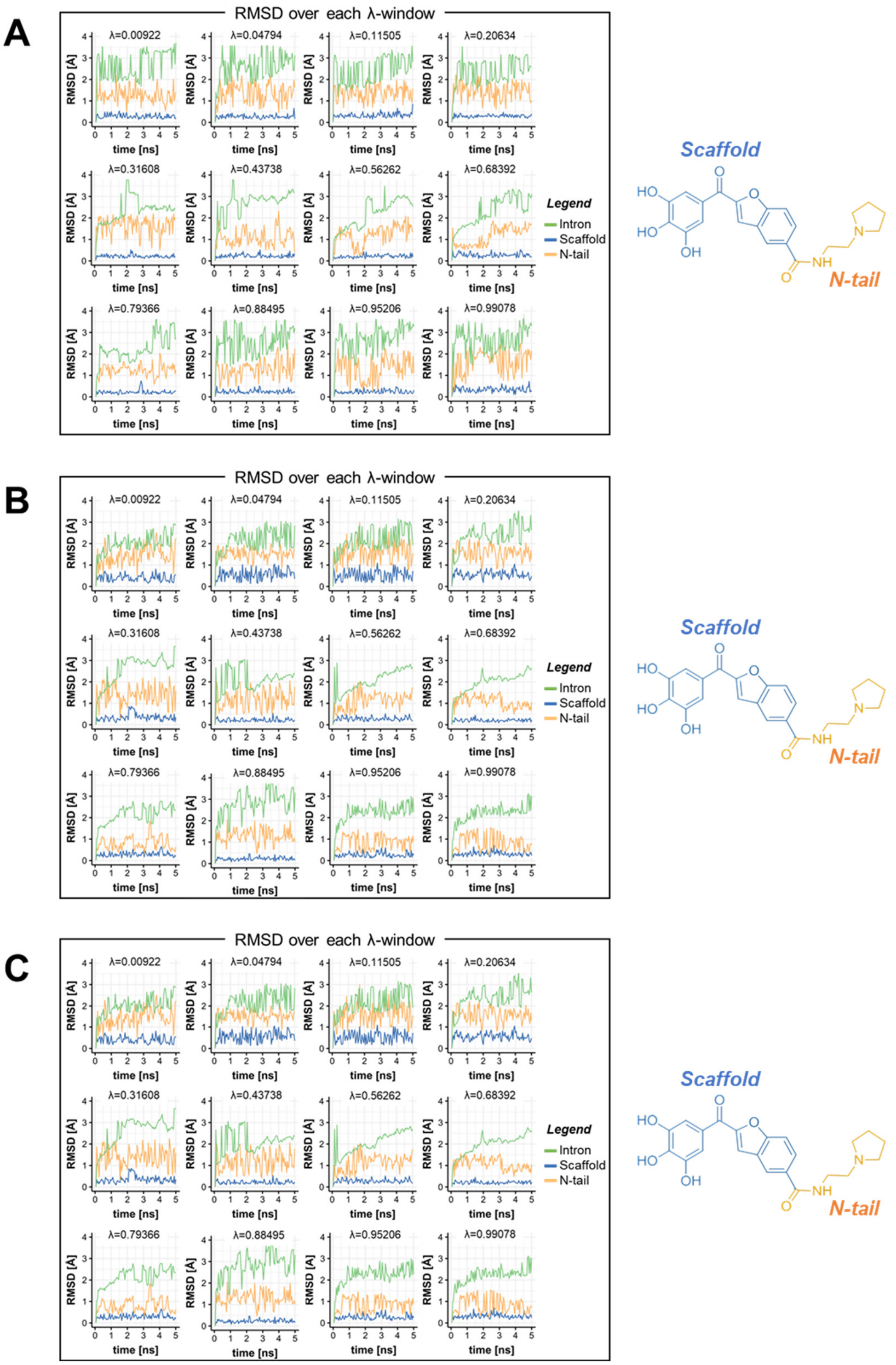

Figure S13. Ligands binding stability during alchemical free energy calculations for compound 8 and intronistat B bound to the intron in complex with the 5'-exon. (A-C) The RMSD values of the intron (green), the intronistat B benzofuran scaffold (blue), and its N-tail (yellow, coloring scheme following that of **Figure 5**), are reported as a function of simulation time at each lambda window, for three simulations replicate. High flexibility is shown by the N-tail but not by the benzofuran scaffold (RMSD<0.5Å) in all windows. This results in a better convergence of the ΔΔG estimates.

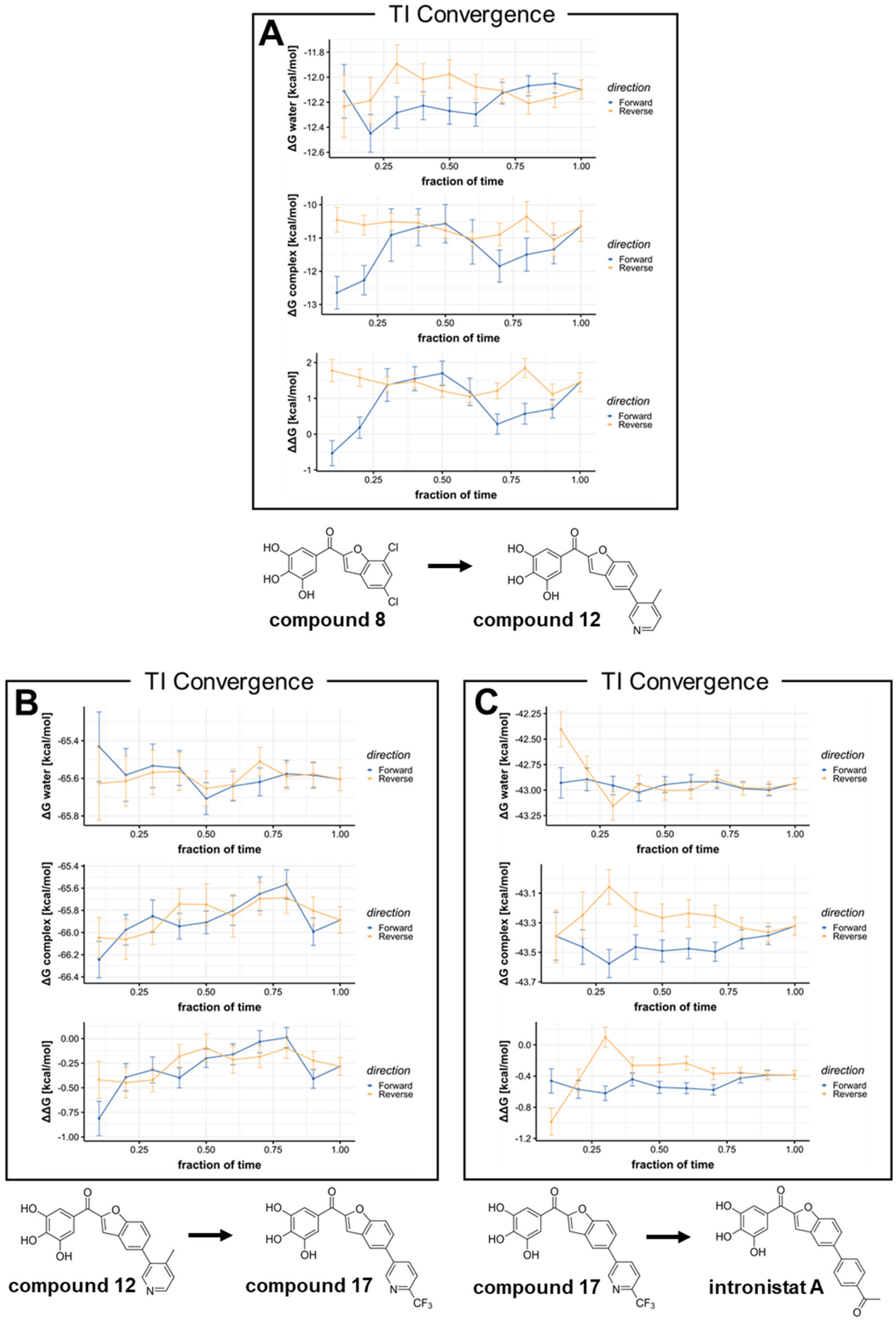

Figure S14. Alchemical free energy calculations for additional intronistat B analogues as bound to the intron in complex with the 5'-exon. The convergence of the forward (blue) and reverse (yellow) estimates of the ΔG of the ligands in water, as bound to the receptor and their ΔΔG is reported for the alchemical transformation of compound 8 and compound 12 **(A)**, compound 12 and compound 17 **(B)**, as well as compound 17 and intronistat A **(C)**. The 2D structure of the compounds involved in the alchemical transformation is reported below each panel.

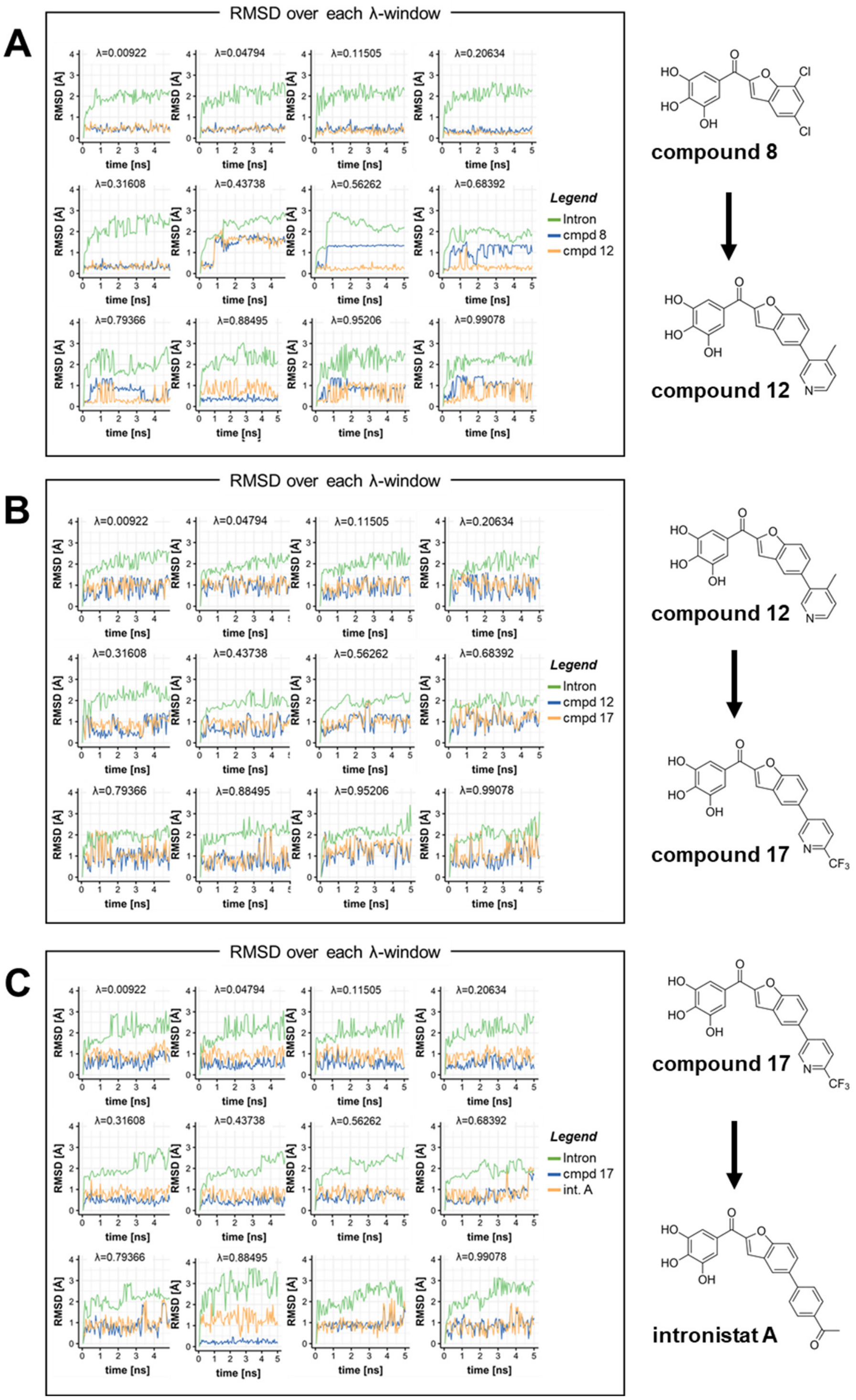

Figure S15. Ligands binding stability during alchemical free energy calculations of intronistat B analogues as bound to the intron in complex with the 5'-exon. A-C) The RMSD values of the intron (green), the intronistat B benzofuran scaffold (blue), and its N-tail (yellow, coloring scheme following that of **Figure 5**), are reported as a function of simulation time at each lambda window, for the alchemical transformation of compound 8 and compound 12 **(A)**, compound 12 and compound 17 **(B)**, as well as of compound 17 and intronistat A **(C)**. The 2D structure of the compounds involved in the alchemical transformation is reported beside each panel.

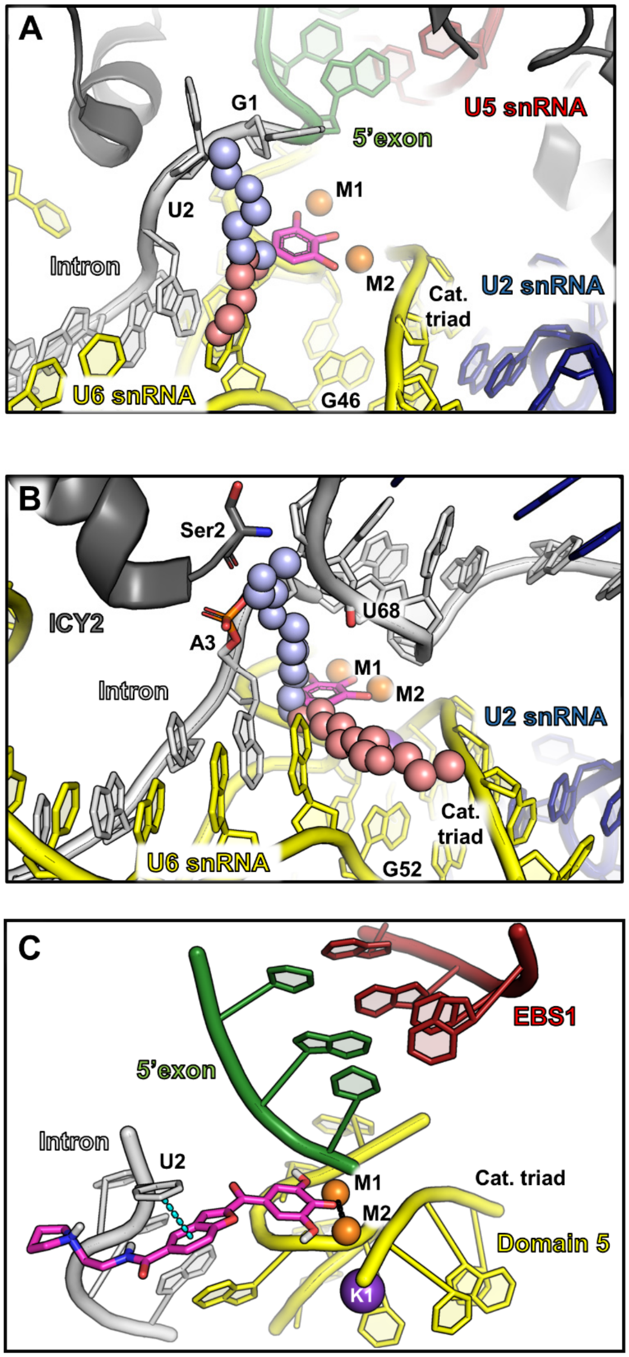

Figure S16. Putative binding modes of intronistat B analogues at the active site of the spliceosome at different stages of catalysis. (A) Intronistat B derivatives bound at catalytic core of the human B* spliceosomal complex (PDB id: 5Z58). **(B)** A similar binding pose can be modeled for intronistat B derivatives when bound to the Ci spliceosomal complex from *S. cerevisiae* (PDB id: 7B9V). Notably, in both cases, a two-metal-ion binding compound similar to intronistat B would locate in proximity of the splice junctions or the intron nucleotides, suggesting that sequence-specific contacts between small molecules and the spliceosomal complex can be possibly engaged upon having anchored its structurally-conserved active site. **(C)** Intronistat B bound at catalytic core of Oi group II intron.

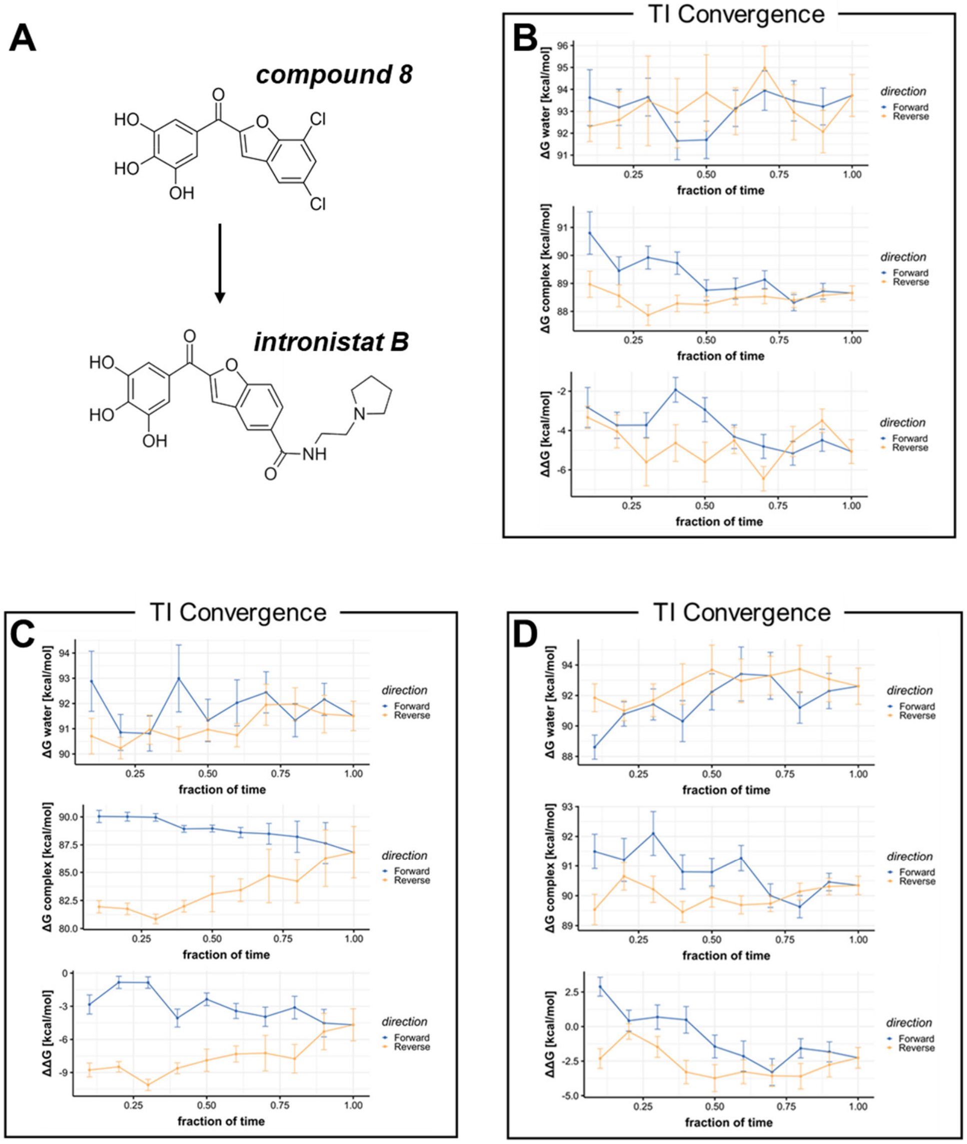

Figure S17. Alchemical free energy calculations for compound 8 and intronistat B bound to the free intron. (A) The 2D structures of the compounds are reported. The convergence of the forward (blue) and reverse (yellow) estimates of the ΔG of the ligands in water and as bound to the receptor, as well their ΔΔG is reported of each of three simulations replica **(B-D)**.

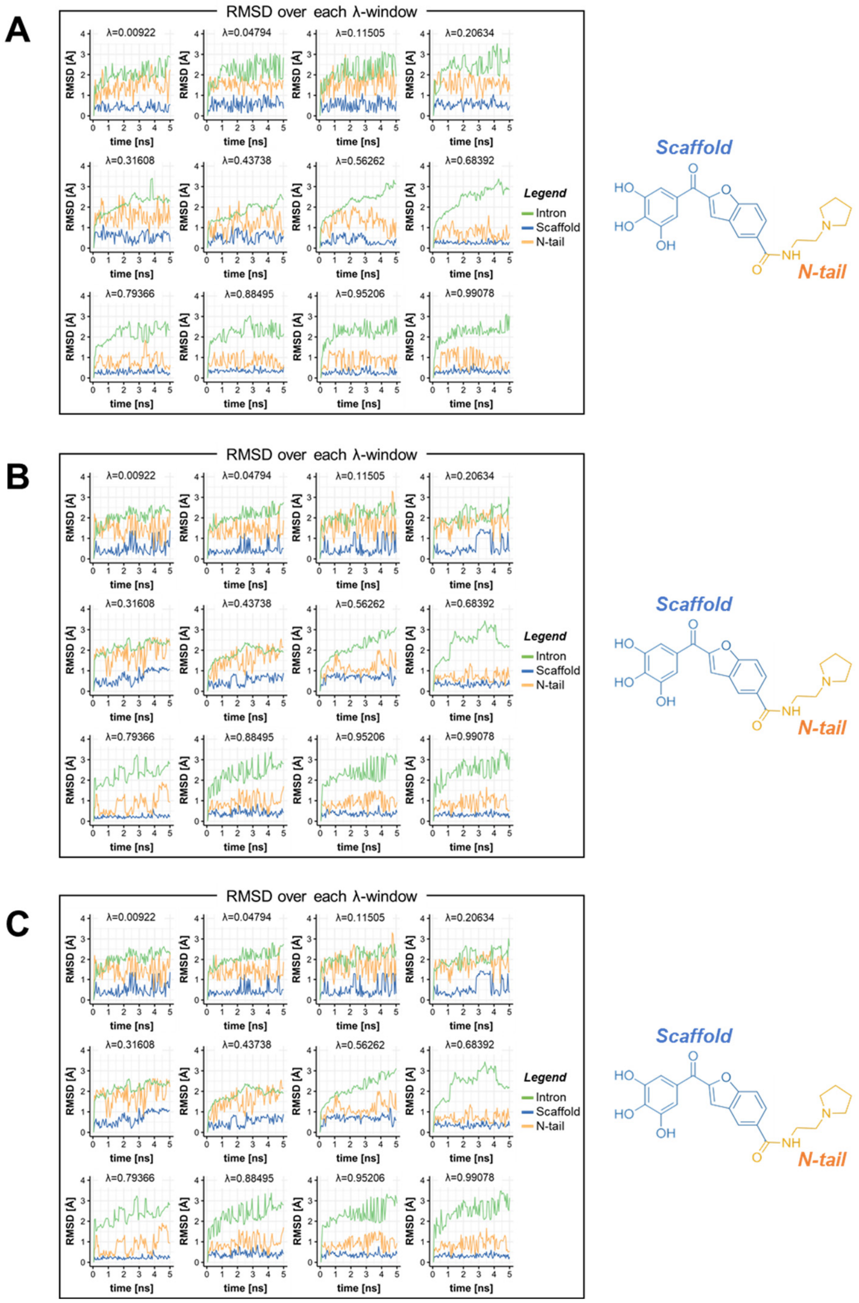

Figure S18. Ligands binding stability during alchemical free energy calculations for compound 8 and intronistat B bound to the free intron. (A-C) The RMSD values of the intron (green), the intronistat B benzofuran scaffold (blue), and its N-tail (yellow, coloring scheme following that of **Figure 5**), are reported as a function of simulation time at each lambda window, for the three simulations replicate. High flexibility is shown by the N-tail and the benzofuran scaffold in several windows, as highlighted by their RMSD fluctuations greater than 2Å and 1Å, respectively. This results in the poor convergence of the ΔΔG estimates.

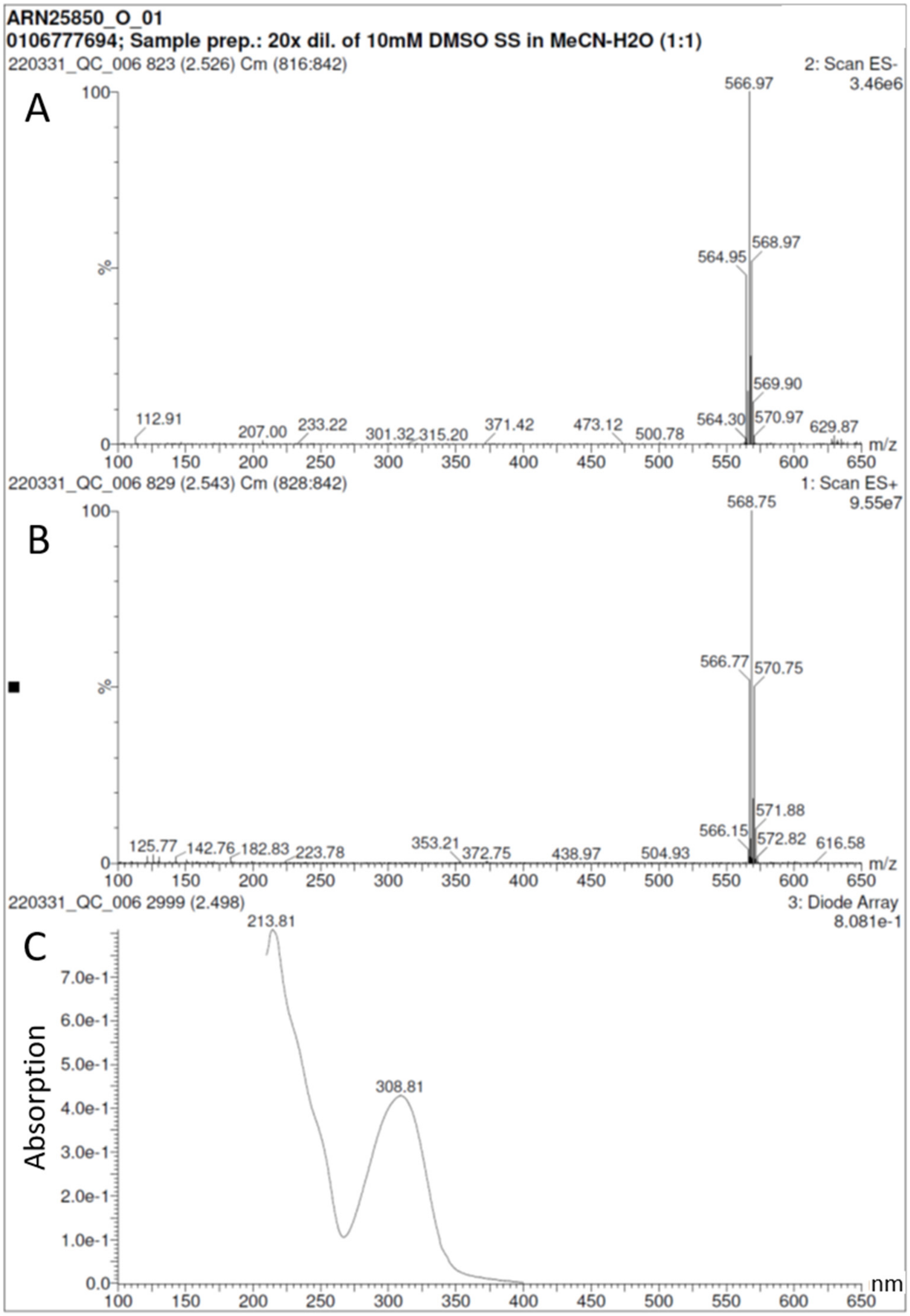

Figure S19. Synthesis of dibromo-intronistat B hydrobromide, ARN25850. UPLC-MS and UV spectra.

1

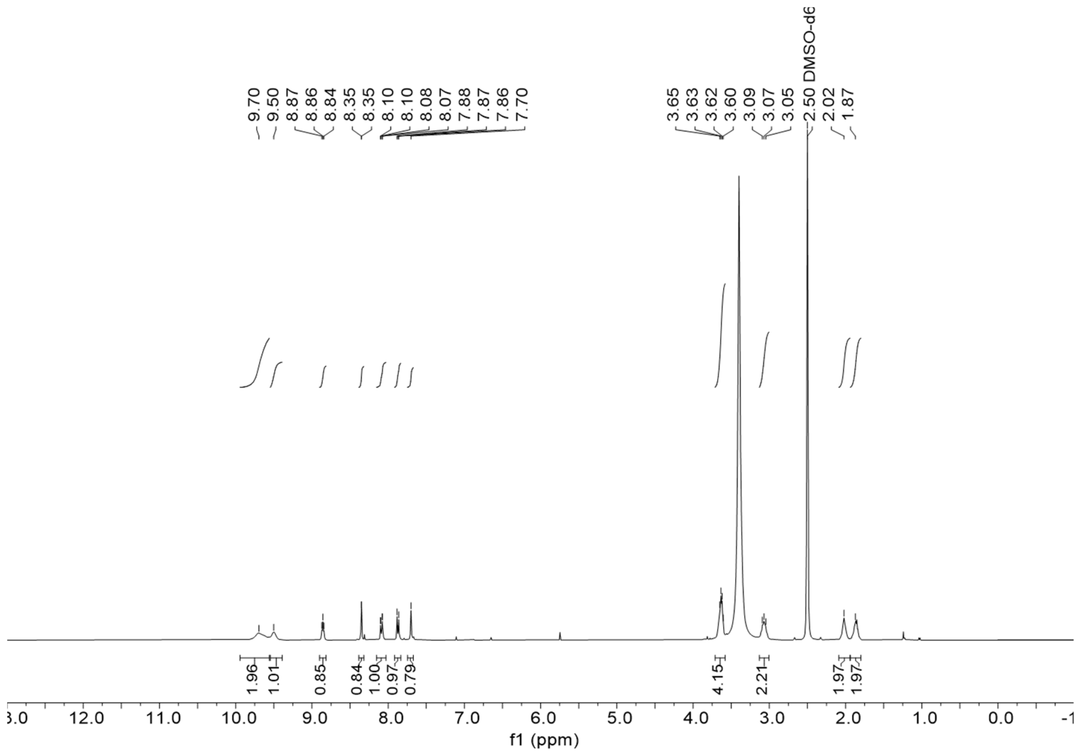

Figure S20. Synthesis of dibromo-intronistat B hydrobromide, ARN25850. ^{1}H-NMR spectrum (DMSO-d_6, 400MHz).

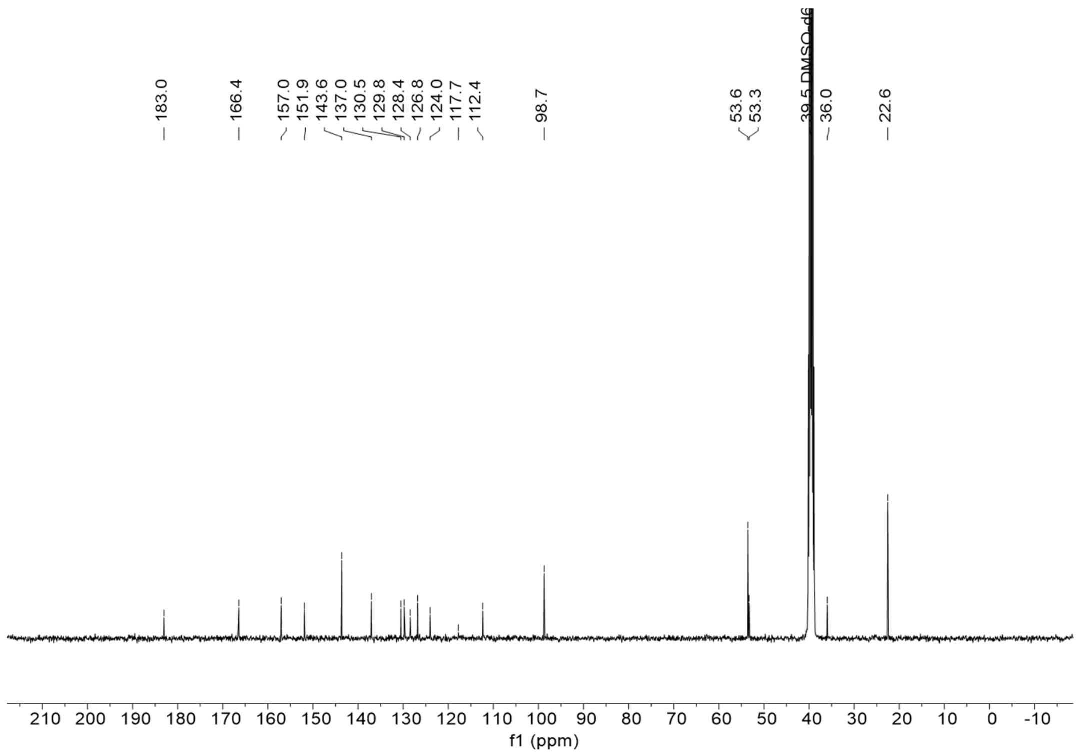

Figure S21. Synthesis of dibromo-intronistat B hydrobromide, ARN25850. ^{13}C-NMR spectrum (DMSO-d_6, 400MHz).

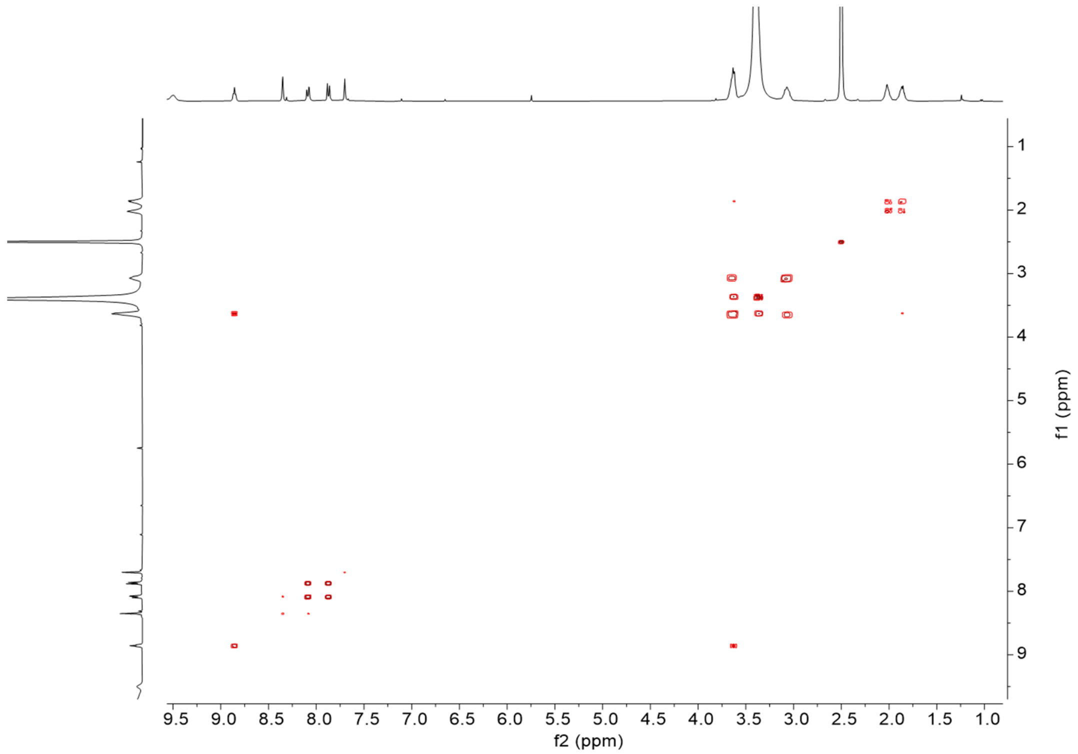

Figure S22. Synthesis of dibromo-intronistat B hydrobromide, ARN25850. COSY spectrum (DMSO-d_6, 400MHz).

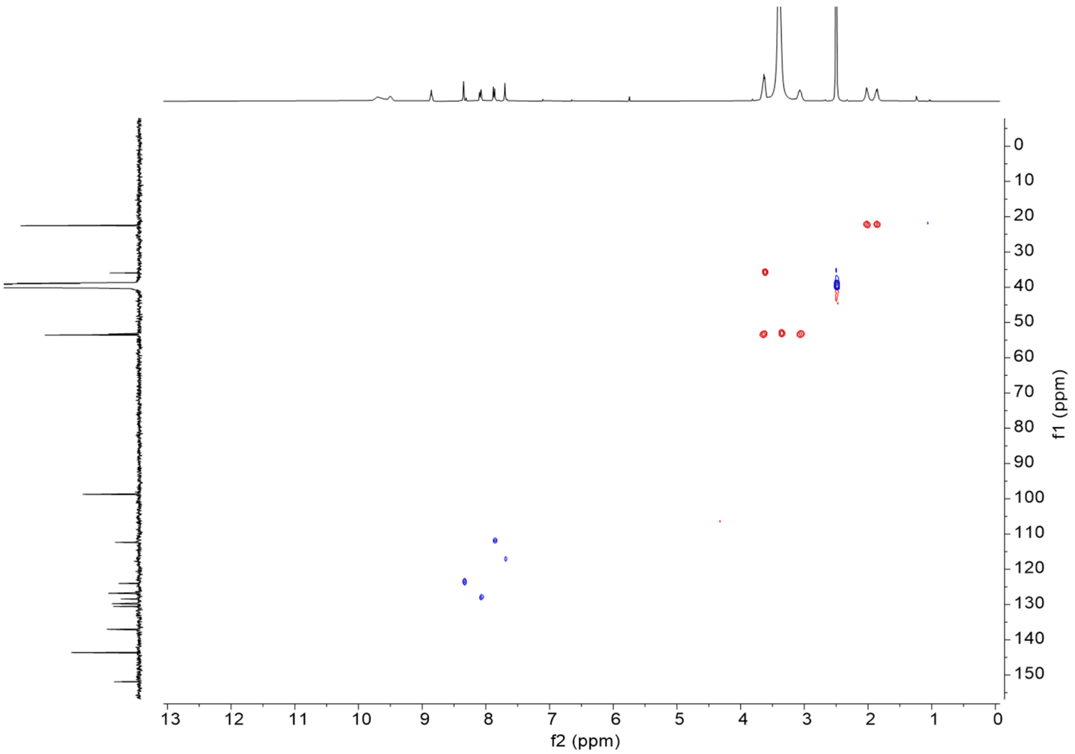

Figure S23. Synthesis of dibromo-intronistat B hydrobromide, ARN25850. HSQC spectrum (DMSO-d_6, 400MHz).

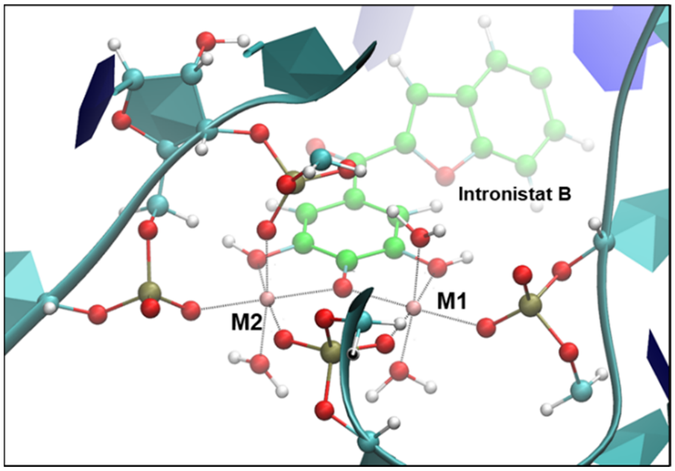

Figure S24. QM/MM partitions of intronistat B in deprotonated form. The figure shows as balls and sticks the atoms of the QM region, which includes the pyrogallol and benzofuran moieties of intronistat B (shown in green) and the magnesium ions (in pink) with their full coordination sphere. Dotted black lines highlight the octahedral coordination around both the magnesium ions. Similar QM/MM partitions were used for the protonated form of Intronistat B and the brominated derivative.

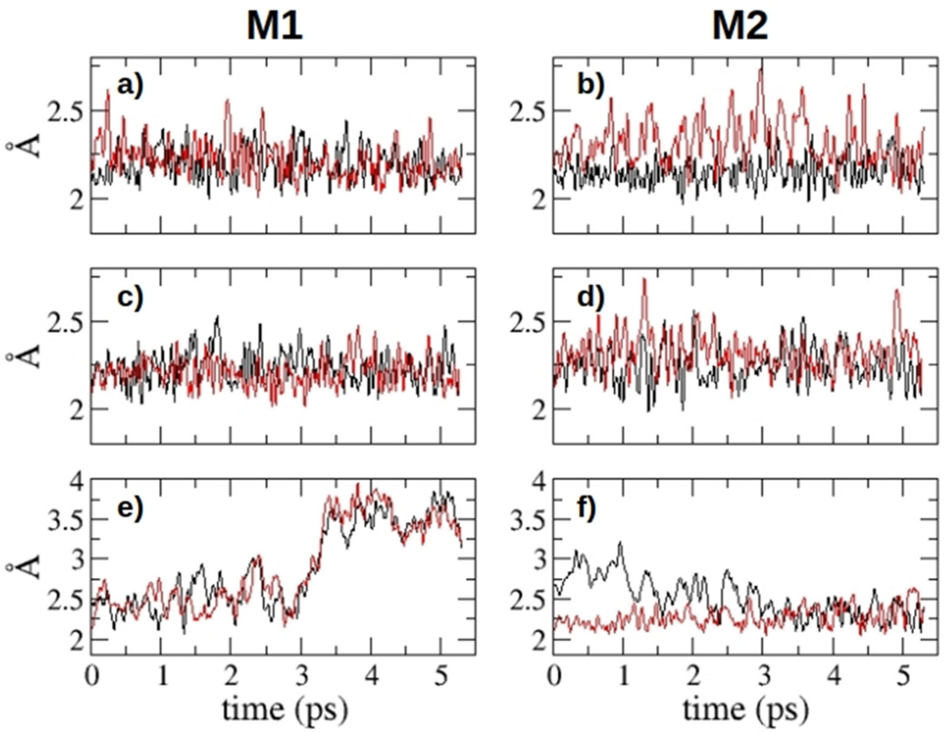

Figure S25. Protonated pyrogallol moiety is incompatible with the Intronistat B binding mode captured by X-ray crystallography. QM/MM simulations of intronistat B in deprotonated form **(A and B)**, the brominated derivative in deprotonated form **(C and D)** and intronistat B in protonated form **(E and F)**. The plots show the distance between the oxygen atoms of the ligands and magnesium ions M1 (left panels) and M2 (right panels). When not protonated, both intronistat B and its brominated derivative steadily coordinates both M1 and M2 catalytic ions **(A-D)**. On the contrary, the protonated form of intronistat B detaches from M1 after ~3 ps simulation **(E)**, while maintaining the binding to M2 **(F)**, resulting in a change of the binding mode of intronistat B to the two-metal ions active site which is incompatible with X-ray crystallography results. This further suggest that intronistat B binds the intron active site when the pyrogallol moiety is deprotonated at para position.

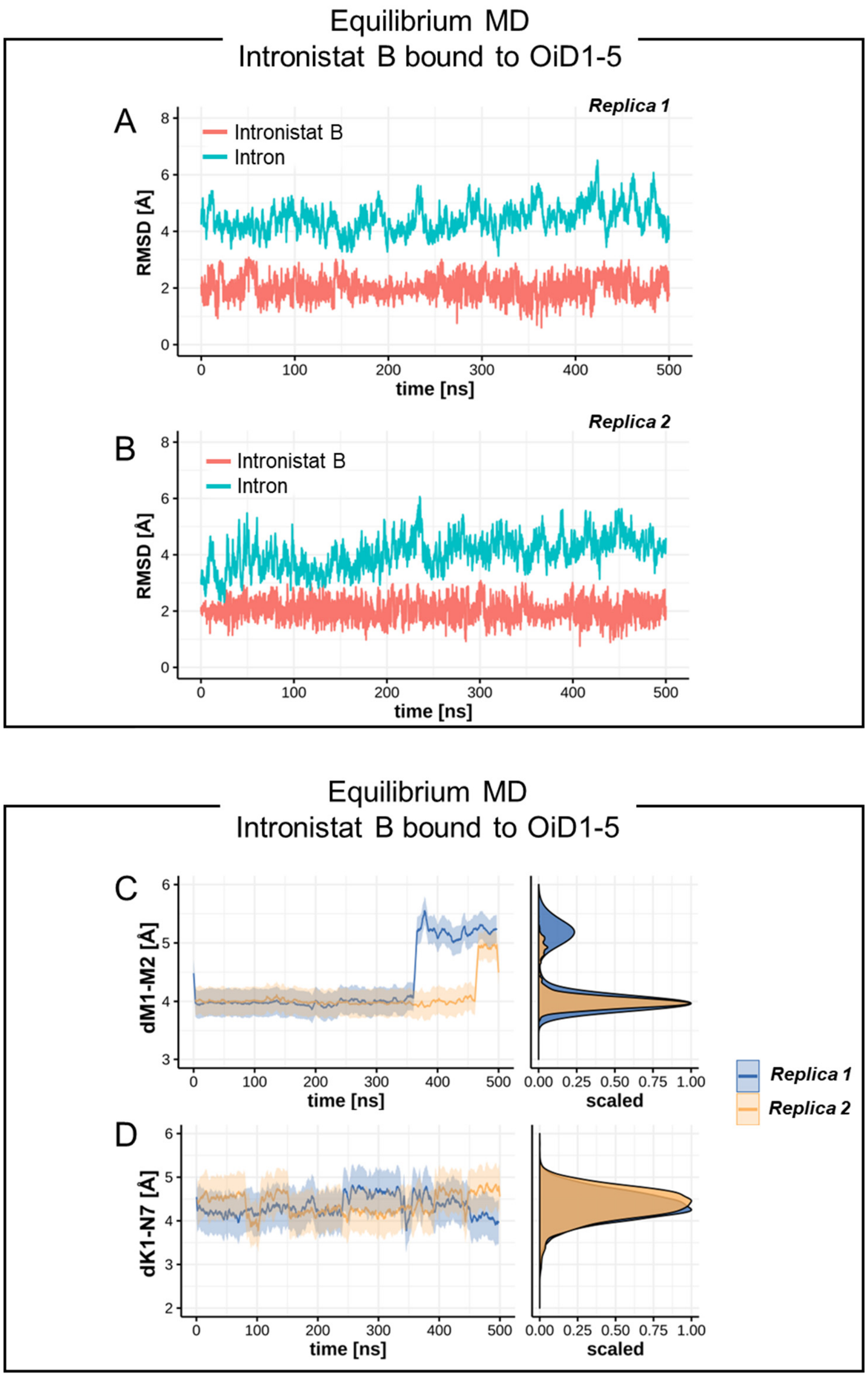

Figure S26. Binding of splicing modulators alters the functional dynamics of intron's catalytic features. The Figure reports MD replicas in support of simulations shown in **Figure 3**.

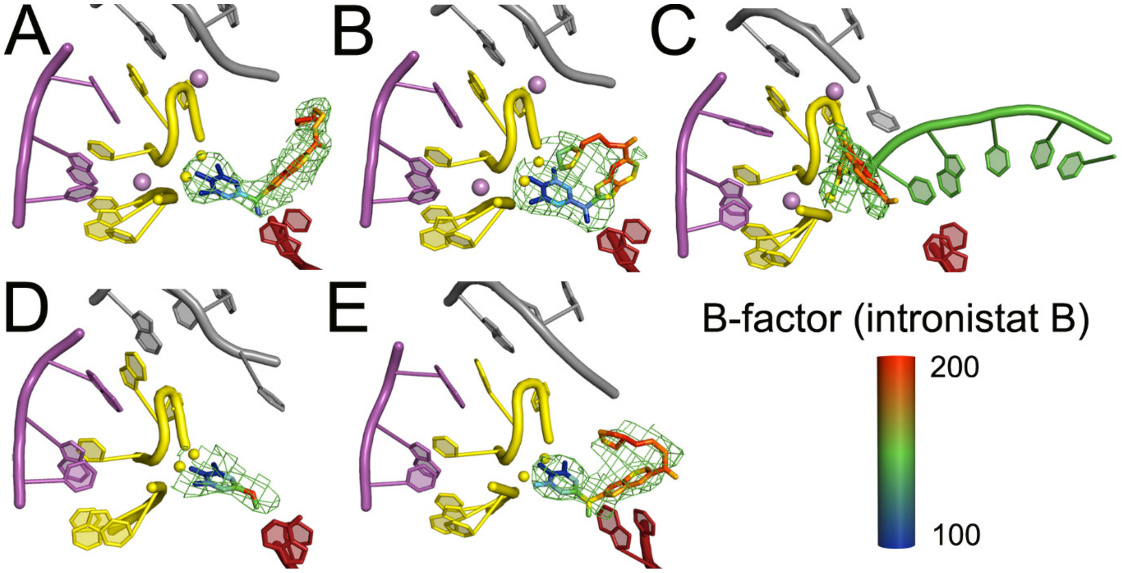

Figure S27. B-factor analysis of intronistat B. (**A**) Crystal structure of OiD1-5 in the presence of Mg^{2+} (yellow spheres), K^+ (purple spheres) and intronistat B. (**B**) Crystal structure of OiD1-5 in the presence of Mg^{2+} (yellow spheres), K^+ (purple spheres), the 5'-exon-like oligonucleotide 5'-AUUUAU-3' and intronistat B after 1h soaking. (**C**) Crystal structure of OiD1-5 in the presence of Mg^{2+} (yellow spheres), K^+ (purple spheres), the 5'-exon-like oligonucleotide 5'-AUUUAU-3', and intronistat B after 2h30' soaking. (**D**) Crystal structure of OiD1-5 in the presence of Mg^{2+} (yellow spheres), Na^+ and intronistat B. (**E**) Crystal structure of OiD1-5 in the presence of Mg^{2+} (yellow spheres), Li^+ and intronistat B. The main active site elements i.e. the J2/3 junction (in magenta), the catalytic triad (in yellow), the 2-nucleotide bulge (in yellow) and the EBS1 site (in firebrick) are shown as cartoons. The F_o−F_c electron density omit map for the structure calculated before modelling intronistat B, contoured at 3σ, is represented as a green mesh. Intronistat B is color-coded by the B-factor values of its atoms (legend on the bottom right corner of the Figure).

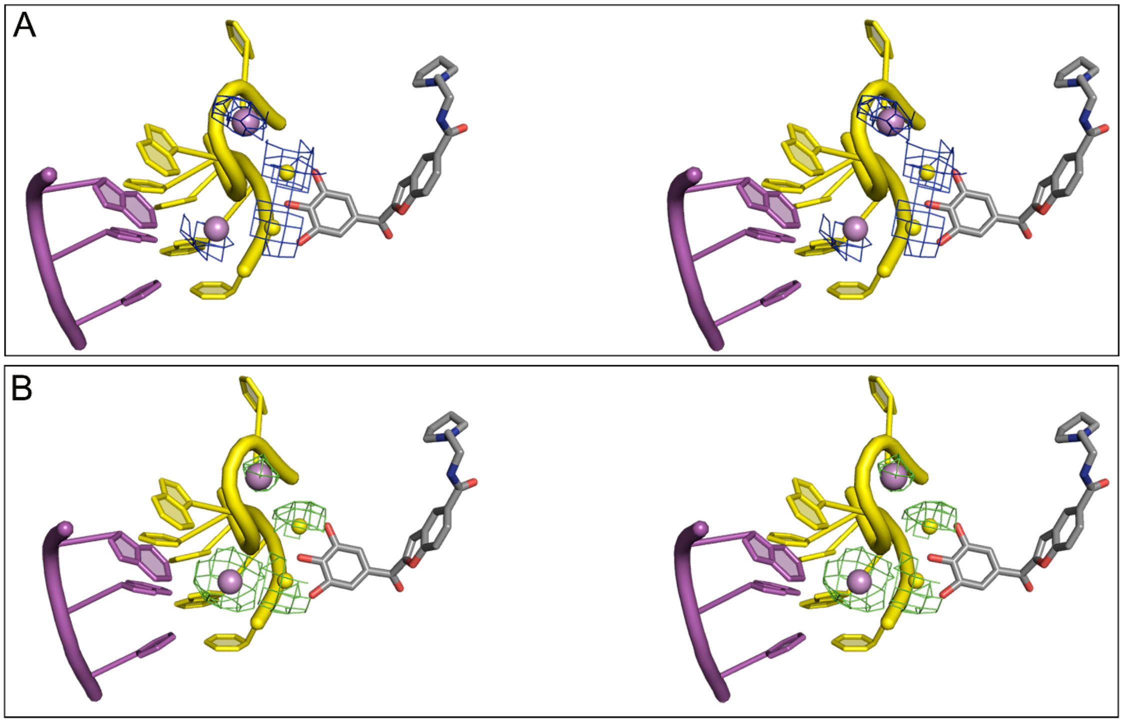

Figure S28. Stereo pair for cross-eyed viewing of the structure of OiD1-5 in the presence of Mg^{2+}, K^{+} and intronistat B. (**A**) Crystal structure of OiD1-5 in the presence of Mg^{2+} (yellow spheres), K^{+} (purple spheres) and intronistat B (grey sticks). The $2F_o{-}F_c$ electron density refined map, contoured at 0.8σ, is represented as a blue mesh. The electron density signal is visible up to 4.0σ for M1, up to 4.5σ for M2, up to 1.6σ for K1, and up to 3.0σ for K2. (**B**) Crystal structure of OiD1-5 in the presence of Mg^{2+} (yellow spheres), K^{+} (purple spheres) and intronistat B (grey sticks). The $F_o{-}F_c$ electron density difference map for the structure calculated before modelling the ions, contoured at 3σ, is represented as a green mesh. The main active site elements, i.e. the J2/3 junction (in magenta), the catalytic triad (in yellow) and the 2-nucleotide bulge (in yellow) are shown as cartoons.

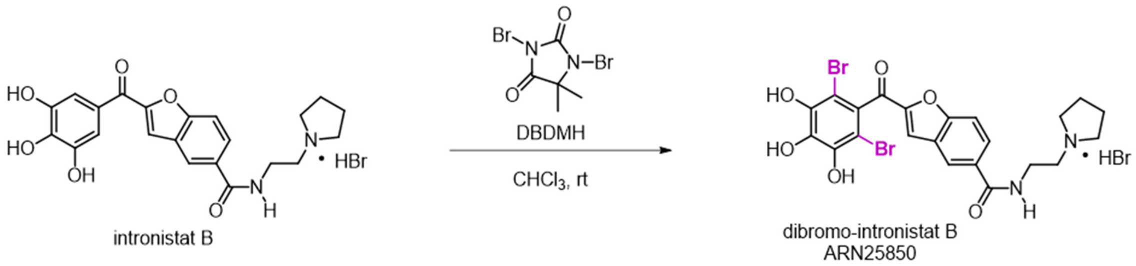

Figure S29. Chemical synthesis of dibromo-intronistat B hydrobromide, ARN25850.

Supplemental Tables

Table S1: Kinetics rate constants. Splicing rate constants of the first (k_1) and second (k_2) steps of splicing of *O. iheyensis* group IIC intron in the presence of all concentrations of intronistat B and the di-brominated intronistat B derivative tested in this study. The total rate constant ($k = k_1 + k_2$) is also reported for easier comparison with previously reported rate constants of the mitochondrial ai5γ intron[3]. N.D. = not determined (under these conditions inhibition is too high to accurately estimate a rate constant). Errors represent standard errors of the mean (s.e.m.) calculated from n = 3 independent experiments.

**[Intronistat B] (μM)**	**k_1 (min^{-1})**	**k_2 (min^{-1})**	**k (min^{-1})**
250	N.D.	N.D.	N.D.
100	0.003 ± 0.001	N.D.	0.003 ± 0.001
50	0.007 ± 0.001	0.001 ± 0.000	0.008 ± 0.001
20	0.004 ± 0.001	0.002 ± 0.000	0.006 ± 0.001
10	0.006 ± 0.000	0.002 ± 0.000	0.008 ± 0.000
1	0.022 ± 0.004	0.021 ± 0.005	0.043 ± 0.007
0.5	0.030 ± 0.001	0.025 ± 0.001	0.055 ± 0.007
0.05	0.042 ± 0.004	0.036 ± 0.003	0.078 ± 0.007
0	0.037 ± 0.002	0.031 ± 0.003	0.068 ± 0.004
**[di-Br-Intronistat B] (μM)**	**k_1 (min^{-1})**	**k_2 (min^{-1})**	**k (min^{-1})**
250	0.006± 0.001	0.006± 0.001	0.012 ± 0.004
100	0.012 ± 0.001	0.009 ± 0.002	0.021 ± 0.003
50	0.019 ± 0.005	0.015 ± 0.005	0.034 ± 0.010
20	0.020 ± 0.004	0.017 ± 0.004	0.037 ± 0.006
10	0.018 ± 0.004	0.013 ± 0.004	0.031 ± 0.007
1	0.028 ± 0.004	0.024 ± 0.004	0.052 ± 0.008
0.5	0.030 ± 0.003	0.023 ± 0.003	0.053 ± 0.006
0	0.037 ± 0.002	0.031 ± 0.003	0.068 ± 0.004

Table S2: X-ray data collection and refinement statistics (molecular replacement).

**Construct**	**OiD1-5**	**OiD1-5**	**Oi5eD1-5**	**Oi5eD1-5**	**OiD1-5**	**OiD1-5**	**OiD1-5**	**OiD1-5**	**OiD1-5**	**OiD1-5**	**OiD1-5**	**OiD1-5**	**OiD1-5**
**Substrate**	**-**	**-**	**-**	**-**	**5'-exon**	**5'-exon**	**5'-exon**	**5'-exon**	**-**	**-**	**-**	**-**	**-**
**Divalent ion**	Mg^{2+}	Mg^{2+}	Ca^{2+}	Mg^{2+}	Mg^{2+}	Mg^{2+}	Mg^{2+}	Mg^{2+}	Mg^{2+}	Mg^{2+}	Mg^{2+}	Mg^{2+}	Mg^{2+}
**Monovalent ion**	K^+	K^+	K^+	K^+	K^+	K^+	K^+	K^+	Na^+	Na^+	Li^+	Li^+	Li^+
**Inhibitor**	**intronistat B**	**ARN25850**	**intronistat B**	**intronistat B**	**-**	**intronistat B**	**ARN25850**	**intronistat B**	**intronistat B**	**ARN25850**		**intronistat B**	**ARN25850**
**Soaking time**						**1h soaking**	**1h soaking**	**2.5h soaking**					
**PDB code**	8OLS	8OLV	8OLW	8OLY	8RUH	8RUI	8RUJ	8OLZ	8OM0	8RUK	8RUL	8RUM	8RUN
**Data Collection**													
Wavelength	0.96	0.92	0.96	0.96	0.96	0.96	0.92	0.96	0.96	0.92	0.96	0.96	0.92
Resolution range[a]	39.7 - 3.0 (3.1 - 3.0)	48.4 - 2.8 (2.9- 2.8)	48. - 4.0 (4.1 - 4.0)	48.0- 3.1 (3.2 - 3.1)	89.0 - 4.5 (4.7 - 4.5)	49.4 - 3.3 (3.4 - 3.3)	49.4 - 3.1 (3.236 - 3.124)	39.8 - 3.3 (3.4 - 3.3)	42.3 - 3.6 (3.7 - 3.6)	48.5 - 4.8 (4.9 - 4.8)	49.1 - 3.6 (3.7 - 3.6)	49.0 - 3.61(3.7 - 3.6)	49.0 - 3.7 (3.9 - 3.7)
Space group	$P 2_1 2_1 2_1$	$P 2_1 2_1 2_1$	$P 2_1 2_1 2_1$	$P 2_1 2_1 2_1$	$P 2_1 2_1 2_1$	$P 2_1 2_1 2_1$	$P 2_1 2_1 2_1$	$P 2_1 2_1 2_1$	$P 2_1 2_1 2_1$	$P 2_1 2_1 2_1$	$P 2_1 2_1 2_1$	$P 2_1 2_1 2_1$	$P 2_1 2_1 2_1$
Cell dimensions:													
a, b, c (Å)	89.0, 95.0, 223.9	90.0, 94.7, 225.0	88.1, 94.1, 222.9	88.5, 94.7, 222.9	90.2 96.5 231.7	89.7 94.9 227.0	89.6 95.1 227.8	87.7, 94.0, 223.4	88.8, 94.3, 223.9	88.8 95.1 225.5	89.2 95.5 224.3	89.1 94.8 224.2	88.9 95.1 223.8
α, β, γ (°)	90, 90, 90	90, 90, 90	90, 90, 90	90, 90, 90	90, 90, 90	90, 90, 90	90, 90, 90	90, 90, 90	90, 90, 90	90, 90, 90	90, 90, 90	90, 90, 90	90, 90, 90
Total reflections[a]	207169 (20770)	582731 (48555)	74878 (21251)	142551 (12794)	76188 (7490)	167823 (8176)	475049 (48091)	183125 (18114)	91376 (8942)	122033 (12579)	142657 (11705)	141761 (11574)	269954 (27242)
Unique reflections[a]	38577 (3799)	46958 (4256)	16065 (1442)	34101 (3245)	12132 (1156)	28279 (2127)	35276 (3419)	28270 (2786)	21631 (2103)	9771 (947)	22529 (2210)	22380 (2183)	20343 (1962)
Multiplicity[a]	5.4 (5.5)	12.4 (11.4)	4.6 (4.8)	4.2 (3.9)	6.3 (6.5)	5.9 (3.8)	13.5 (14.1)	6.5 (6.5)	4.2 (4.3)	12.5 (13.3)	6.3 (5.3)	6.3 (5.3)	13.3 (13.9)
Completeness (%)[a]	99.31 (99.48)	97.90 (79.41)	97.77 (85.51)	98.69 (93.76)	99.70 (98.89)	94.25 (66.80)	98.96 (91.55)	99.46 (98.71)	96.82 (96.77)	99.79 (99.89)	99.87 (99.73)	99.81 (99.32)	99.68 (97.90)
Mean I/sigma(I)[a]	18.58 (0.92)	11.97 (0.29)	5.8 (0.9)	10.57 (0.67)	11.08 (1.20)	13.47 (0.62)	11.90 (0.37)	4.57 (0.47)	12.17 (0.71)	12.14 (2.31)	13.08 (1.45)	12.57 (1.22)	16.15 (1.44)
**Refinement**													
Wilson B-factor	123.04	114.75	215.82	132.56	235.74	154.18	137.33	137.98	202.19	186.07	152.42	161.23	173.29
R-merge[a]	0.044 (2.0)	0.11 (5.8)	0.097 (2.1)	0.069 (1.9)	0.054 (1.3)	0.057 (1.7)	0.12 (4.8)	0.21 (2.7)	0.043 (2.3)	0.14 (1.4)	0.08 (1.1)	0.07 (1.2)	0.09 (2.0)
R-meas[a]	0.049 (2.2)	0.115 (6.0)	0.110 (2.4)	0.080 (2.2)	0.060 (1.4)	0.063 (1.9)	0.12 (4.9)	0.23 (3.0)	0.049 (2.6)	0.15 (1.4)	0.09 (1.2)	0.08 (1.3)	0.09 (2.1)
R-pim[a]	0.021 (0.89)	0.034 (1.73)	0.050 (1.08)	0.040 (1.10)	0.026 (0.55)	0.025 (0.91)	0.034 (1.32)	0.094 (1.16)	0.023 (1.21)	0.043 (0.39)	0.036 (0.53)	0.032 (0.56)	0.025 (0.57)
$CC_{1/2}$[a]	1.0 (0.35)	1.0 (0.083)	1.0 (0.28)	1.0 (0.22)	0.99 (0.57)	0.99 (0.46)	0.99 (0.32)	0.99 (0.32)	1.0 (0.20)	1.0 (0.8)	1.0 (0.7)	1.0 (0.5)	1.0 (0.6)
No. reflections[a]	38568 (3799)	46400 (3718)	15922 (1346)	34019 (3187)	12123 (1155)	28051 (1956)	34949 (3154)	28160 (2754)	21611 (2099)	9766 (947)	22513 (2205)	22369 (2181)	20327 (1959)
R-work[a]	0.19 (0.43)	0.21 (0.75)	0.22 (0.40)	0.20 (0.40)	0.18 (0.25)	0.18 (0.41)	0.20 (0.46)	0.20 (0.41)	0.23 (0.39)	0.18 (0.22)	0.17 (0.37)	0.18 (0.37)	0.17 (0.34)
R-free[a,b]	0.25 (0.41)	0.24 (0.69)	0.25 (0.41)	0.23 (0.37)	0.25 (0.32)	0.23 (0.37)	0.24 (0.50)	0.27 (0.42)	0.28 (0.40)	0.24 (0.35)	0.24 (0.52)	0.24 (0.39)	0.23 (0.34)

No. atoms:													
RNA	8349	8438	8497	8475	8440	8341	8349	8455	8349	8270	8428	8405	8416
ligand/ion	145	115	21	107	62	67	71	112	45	46	84	69	90
water	13	11	11	49	45	40	63	15	0	8	57	89	61
B-factor:													
RNA	144	126	222	146	212	162	154	146	226	175	145	157	166
ligand/ion	174	160	208	125	149	177	171	143	199	223	128	153	182
water	104	91	168	119	95	115	124	82		66	95	108	113
RMSD:													
bond length (Å)	0.042	0.018	0.011	0.011	0.009	0.031	0.017	0.030	0.042	0.017	0.009	0.016	0.017
bond angles (°)	2.15	1.89	2.22	1.69	1.71	2.50	2.70	1.90	2.65	1.86	2.28	2.37	2.36

One single crystal was used to collect each data set.
[a]Values in parentheses are for the highest-resolution shell.
[b]R-free was calculated using 5% of the total reflections.

Supplemental References

1 Manigrasso, J. *et al.* Visualizing group II intron dynamics between the first and second steps of splicing. *Nature communications* **11**, 2837 (2020). https://doi.org:10.1038/s41467-020-16741-4

2 Marcia, M. & Pyle, A. M. Visualizing group II intron catalysis through the stages of splicing. *Cell* **151**, 497-507 (2012). https://doi.org:10.1016/j.cell.2012.09.033

3 Fedorova, O. *et al.* Small molecules that target group II introns are potent antifungal agents. *Nature chemical biology* **14**, 1073-1078 (2018). https://doi.org:10.1038/s41589-018-0142-0
