# Supplementary material for: Targeting the conserved active site of splicing machines with specific and selective small molecule modulators
Source: Nat Commun. 2024 Jun 19;15:4980. doi: 10.1038/s41467-024-48697-0 (PMC11187226; doi:10.1038/s41467-024-48697-0)
Supplement: Supplementary file 3 — Reporting Summary [file 41467_2024_48697_MOESM3_ESM.pdf]

nature portfolio

Corresponding author(s): Marco De Vivo, Marco Marcia

Last updated by author(s): Apr 24, 2024

Reporting Summary

Nature Portfolio wishes to improve the reproducibility of the work that we publish. This form provides structure for consistency and transparency in reporting. For further information on Nature Portfolio policies, see our Editorial Policies and the Editorial Policy Checklist.

Statistics

For all statistical analyses, confirm that the following items are present in the figure legend, table legend, main text, or Methods section.

n/a	Confirmed	
☐	☒	The exact sample size (n) for each experimental group/condition, given as a discrete number and unit of measurement
☐	☒	A statement on whether measurements were taken from distinct samples or whether the same sample was measured repeatedly
☒	☐	The statistical test(s) used AND whether they are one- or two-sided<br>*Only common tests should be described solely by name; describe more complex techniques in the Methods section.*
☒	☐	A description of all covariates tested
☒	☐	A description of any assumptions or corrections, such as tests of normality and adjustment for multiple comparisons
☐	☒	A full description of the statistical parameters including central tendency (e.g. means) or other basic estimates (e.g. regression coefficient) AND variation (e.g. standard deviation) or associated estimates of uncertainty (e.g. confidence intervals)
☒	☐	For null hypothesis testing, the test statistic (e.g. F, t, r) with confidence intervals, effect sizes, degrees of freedom and P value noted<br>*Give P values as exact values whenever suitable.*
☒	☐	For Bayesian analysis, information on the choice of priors and Markov chain Monte Carlo settings
☒	☐	For hierarchical and complex designs, identification of the appropriate level for tests and full reporting of outcomes
☒	☐	Estimates of effect sizes (e.g. Cohen's d, Pearson's r), indicating how they were calculated

Our web collection on statistics for biologists contains articles on many of the points above.

Software and code

Policy information about availability of computer code

Data collection	Automatic data processing pipelines (ID30-A1 and ID30B beamlines, European Synchrotron Radiation Facility; https://www.esrf.fr/UsersAndScience/Experiments/MX/How_to_use_our_beamlines/Run_Your_Experiment/automatic-data-processing); AMBER22 (Salomon-Ferrer et al., 2013; Lee et al., 2017), QUICK code (Cruzeiro et al., 2021; Manathunga et al., 2020), SANDER and AmberTools (Case et al., 2023; Karplus, 2014; Levitt, 2014); MolMovDB (Krebs, 2000); MicroCal PEAQ-ITC Analysis Software v 1.40 (Malvern, 2014), OctetRED96e Data analysis HT v11.1 (Sartorius)
Data analysis	GraphPad Prism v 6 (GraphPad Software); XDS v. March 30th 2013 (Kabsch, 1993); Phenix v 1.14-3260 (Adams, 2010); Refmac5 v 5.7.0032 (Murshudov, 1997); Coot v 0.8.9.2 (Emsley, 2004); PyMOL v 1.8.0.4 (Schrodinger LLC); CCP4 v 7.0 (Collaborative Computational Programme number 4, 1994); Plumed v 2.3 (Bonomi, 2009 and Branduardi, 2012).

For manuscripts utilizing custom algorithms or software that are central to the research but not yet described in published literature, software must be made available to editors and reviewers. We strongly encourage code deposition in a community repository (e.g. GitHub). See the Nature Portfolio guidelines for submitting code & software for further information.

Data

Policy information about availability of data

All manuscripts must include a data availability statement. This statement should provide the following information, where applicable:

- Accession codes, unique identifiers, or web links for publicly available datasets
- A description of any restrictions on data availability
- For clinical datasets or third party data, please ensure that the statement adheres to our policy

Coordinates and structure factors have been deposited in the Protein Data Bank under accession codes 8OLS, 8OLV, 8OLW, 8OLY, 8OLZ, 8OM0, 8RUH, 8RUI, 8RUJ, 8RUK, 8RUL, 8RUM, and 8RUN.
Coordinates of previously published PDB files 4E8M and 4FAR, which we have used as initial models for molecular replacement calculations, are openly accessible from the PDB, as described in this previous publication: Marcia and Pyle, Cell, 2012 (DOI: 10.1016/j.cell.2012.09.033)

Research involving human participants, their data, or biological material

Policy information about studies with human participants or human data. See also policy information about sex, gender (identity/presentation), and sexual orientation and race, ethnicity and racism.

Reporting on sex and gender	N/A
Reporting on race, ethnicity, or other socially relevant groupings	N/A
Population characteristics	N/A
Recruitment	N/A
Ethics oversight	N/A

Note that full information on the approval of the study protocol must also be provided in the manuscript.

Field-specific reporting

Please select the one below that is the best fit for your research. If you are not sure, read the appropriate sections before making your selection.

☒ Life sciences ☐ Behavioural & social sciences ☐ Ecological, evolutionary & environmental sciences

For a reference copy of the document with all sections, see nature.com/documents/nr-reporting-summary-flat.pdf

Life sciences study design

All studies must disclose on these points even when the disclosure is negative.

Sample size	All enzymatic assays were performed at least in triplicates. We have chosen this sample size for our enzymatic experiments because such measurements allow for accurate estimation of standard errors and so that our study is in agreement with previous literature in the field of splicing, i.e. Marcia and Pyle, Cell, 2012 (DOI: 10.1016/j.cell.2012.09.033) and Manigrasso et al, Nature Communications, 2021 (DOI: 10.1038/s41467-020-16741-4). All equilibrium MD simulations were performed in 2 or 3 independent replicas. This amount of calculations was chosen to cumulate a total of at least 1 microsecond simulation time per molecular system. All alchemical free energy calculations were performed with twelve lambda-window simulations, 5ns each. The ITC experiment was performed in triplicate. The BLI experiment was performed in duplicate. We have chosen this sample size for our ITC and BLI experiments because such measurements allow for accurate estimation of standard errors.
Data exclusions	No data were excluded from the analysis.
Replication	All kinetics could be reproduced successfully in at least three independent experiments. 2-3 MD simulations were performed under each condition, as indicated in the text and figures, and simulation results were also reliably reproducible.
Randomization	Randomization is not applicable to our study, because our study is based on structural models and is not conceived in the way other experiments are, where randomization is used as a contrai. The study design could not be randomized as analysis of simulations and experimental results required knowledge of the identity of each sample.
Blinding	Blinding is not applicable to our study, because our study presents crystal structures and computational simulations of crystal structures. Thus, it does not require blinding as a contrai, unlike other type of experiments. There is no way to crystallize a molecule or to run MD simulations accounting for blinding. Blinding does not apply to crystallographic analysis, enzymatic assays, or MD simulations as the identity of the samples are known a priori.

Reporting for specific materials, systems and methods

We require information from authors about some types of materials, experimental systems and methods used in many studies. Here, indicate whether each material, system or method listed is relevant to your study. If you are not sure if a list item applies to your research, read the appropriate section before selecting a response.

Materials & experimental systems

n/a	Involved in the study
☒	☐ Antibodies
☒	☐ Eukaryotic cell lines
☒	☐ Palaeontology and archaeology
☒	☐ Animals and other organisms
☒	☐ Clinical data
☒	☐ Dual use research of concern
☒	☐ Plants

Methods

n/a	Involved in the study
☒	☐ ChIP-seq
☒	☐ Flow cytometry
☒	☐ MRI-based neuroimaging
